# Experiencing social connection: A qualitative study of mothers of nonspeaking autistic children

**DOI:** 10.1371/journal.pone.0242661

**Published:** 2020-11-25

**Authors:** Vikram K. Jaswal, Janette Dinishak, Christine Stephan, Nameera Akhtar

**Affiliations:** 1 Department of Psychology, University of Virginia, Charlottesville, Virginia, United States of America; 2 Department of Philosophy, University of California, Santa Cruz, California, United States of America; 3 Harrisonburg, Virginia, United States of America; 4 Department of Psychology, University of California, Santa Cruz, California, United States of America; Jiangsu Normal University, CHINA

## Abstract

Autistic children do not consistently show conventional signs of social engagement, which some have interpreted to mean that they are not interested in connecting with other people. If someone does not act like they are interested in connecting with you, it may make it difficult to feel connected to them. And yet, some parents report feeling strongly connected to their autistic children. We conducted phenomenological interviews with 13 mothers to understand how they experienced connection with their 5- to 14-year-old nonspeaking autistic children. Mothers of nonspeaking autistic children represent a unique group in which to study connection because their children both may not seem interested in connecting with them *and* have limited ability to communicate effectively using speech, a common way people connect with each other. The mothers in this study interpreted a range of child behaviors—some unconventional, but many conventional—as signs that their children were interested in connecting with them, (re)framed child behaviors that could undermine connection as caused by factors unrelated to the relationship, and expressed several convictions that may help build and sustain connection in the face of uncertainty about the meaning of their children’s behavior. Even though their autistic children may not consistently act in conventional socially oriented ways, these mothers reported perceiving their children’s behavior as embedded within an emotionally reciprocal relationship.

## Introduction

Autistic children behave in ways that are different from non-autistic children. For example, compared to non-autistic children, they are less likely to make eye contact [1], respond to their name [2], or look where someone points [3]. Engaging in these kinds of behaviors is conventionally assumed to reflect social interest [4]—at least in many Western cultures [5]. As a result, children who do not engage in these kinds of behaviors (or who do so inconsistently) can seem to non-autistic observers like they are socially uninterested or withdrawn [6,7].

Indeed, some parents report feeling isolated from their autistic child, in part because they interpret the child’s apparently socially uninterested behavior to mean that the child *is* socially uninterested or even resistant to the parent’s attempts to connect with them [8]. Certainly, some scientists and practitioners seem to interpret autistic children’s behavior in this way. For example, Wimpory, Hobson, Williams, and Nash [9] suggested that “autism involves ‘basic’ impairments in forms of person-to-person interpersonal relatedness” (p. 531). One psychologist who ran a support group for mothers of autistic children recently claimed that “[autistic] children are really unable to be in a reciprocal relationship and the moms don’t really experience the love that comes back from a child—the bonding is mitigated” [10].

And yet, some parents report seeing in their autistic children’s behavior a profound desire to connect with them and with other people. This desire may not always be shown in conventional ways, but these parents report recognizing it nonetheless. For example, in her memoir, Senator [11] explained that she one day realized that her autistic son’s inexplicable laughter—a behavior not conventionally interpreted as a bid for connection—was one way he attempted to connect with members of his family:

“Natty, what is so funny? What is so funny?” I asked playfully, tickling him and poking his ribs. He kept laughing, but now it was in response to my actions and my voice. He was so adorable just then and I felt so much love for him. And then in a few moments, I noticed his laughter dying down in a natural way. He was looking at me warmly. Now my throat was burning—this had cracked me wide open. *Oh my God*, I thought. *He really does it to connect with us*. *He just doesn’t know how*, *other than to annoy us*. I knew this was so because of the way Nat had stopped laughing naturally and on his own. This revelation permanently shifted how I viewed Nat’s difficult behaviors thereafter, allowing me to see more of the person inside and to relate to him in a more satisfying way. (pp. 194–5, emphasis in original).

The study reported here, inspired by this kind of example, asks mothers who say they feel connected to their autistic children to reflect on why they feel that way. This is a very different approach to studying social behavior than is traditionally taken in research on autism or development. As Jaswal and Akhtar [12] pointed out, researchers typically look for particular behaviors that are assumed to be universal indicators of social interest—eye gaze, pointing, and so on—and make inferences about children’s social interest on the basis of how often they engage in those behaviors. In this study, we depart from this tradition by asking mothers to reflect on the behaviors *they* interpret as relevant to social connection (see also [13]).

Before beginning, we need to address how we are using the phrase “social connection” in this study. Social connection is a rich, complex, and varied experiential phenomenon. Questionnaires that ostensibly measure the construct have used items like, “How close do you feel to this person? How connected do you feel to this person? How comforted do you feel by this person? How much does this person understand the way you feel about things?” [14]. Our goal in this study was to understand how our interviewees experienced what they consider to be connection, and so we did not define it for them and will not do so here. In the Results section, however, we will describe some of the characteristics of connection that the mothers highlighted as they explained their experience of it.

In the following sections, we describe why studying social connection between mothers and their autistic children is important. Then we consider how mothers’ “rich interpretation” of behavior may contribute to feelings of connection. Finally, we explain our focus on mothers of autistic children who have limited ability to use speech.

### Why studying social connection matters

Autistic people tend to be more socially isolated than non-autistic people. For example, autistic young adults are less likely to interact with friends than are their non-autistic peers who have other disabilities [15]. Social isolation may not be a problem if it is freely chosen [16]. But for many autistic people, it is not a choice: Both autistic children and adults report higher levels of loneliness compared to non-autistic peers [17], and loneliness in autistic adults has been linked to depression, self-injury, suicidal ideation, and suicidality [18]. Some of the social isolation stems from non-autistic people’s avoidance of people who behave in the unusual ways associated with autism [19]. But some social isolation can also be attributed to non-autistic people misinterpreting autistic people’s behavior as indicating that they are not interested in social interaction [12].

Given this backdrop, understanding what child behaviors mothers interpret as relevant to connection is important for at least two reasons. First, it can provide insight into the variety of ways that social interest can be perceived. As noted, autistic children are frequently thought to be socially uninterested, which could make it difficult for others to engage with them. Investigating how some mothers perceive their children as socially interested may help provide others with the tools to perceive (and act upon) social interest even when it is not shown in expected ways, which could help to reduce the social isolation reported by many autistic people.

Second, understanding how mothers experience connection with their autistic children could be important to efforts to reduce parenting stress. Mothers of autistic children report feeling as close to their children as mothers of non-autistic children (for example, see [20]; but see [8]), but having a child who does not seem socially connected is associated with higher levels of parenting stress [21,22]. For example, in Davis and Carter [21], one measure of social relatedness in toddlers was a parent questionnaire that included items such as, “Likes being cuddled, hugged or kissed by loved ones.” Mothers who perceived their children as more socially related on this questionnaire reported lower levels of parenting stress compared to mothers who perceived their children as less socially related. The causal direction of this association is not certain, but an intriguing possibility is that providing mothers with the tools to perceive social relatedness in their children could contribute to efforts to reduce parenting stress.

### Rich interpretations of behavior

Developmental psychologists use the phrase “rich interpretation” to refer to interpretations of child behavior that the child cannot confirm themselves and which are often suggestive of some underlying competence. Parents of non-autistic infants and children routinely engage in rich interpretation. Indeed, it is thought to play an important role in the emergence of increasingly sophisticated communicative, cognitive, and social reasoning abilities [23,24]: Acting as if a child’s behavior is indicative of a particular competence can create the conditions for the child to learn or develop that competence [25,26]. For example, the frequency with which mothers in Western cultures elaborate on their infants’ babbling—responding to it as if it were meaningful—predicts increases in their infants’ vocal production [27].

In this study and adapting the use from developmental psychology, we use “rich interpretation” to refer to the mothers’ practice of imputing meaning to behavior that goes beyond that particular behavior itself—meaning informed by the observer’s perspective, history with the actor, and understanding of the context. Our use of “rich interpretation” thus seems consistent with Hart’s [28] notion of “parents as translators” or the autistic self-advocate Sinclair’s [29] notion of “parents as interpreters” for their autistic children.

Unless it is explicitly stated, a desire for social connection cannot be measured directly; it is inferred on the basis of behavior. For Senator [11], who was quoted earlier, the particular conditions and sequence in which her son laughed and looked at her led her to conclude that his laughter was a bid for social connection. Her interpretation was made in the context of beliefs about her son’s personality, knowledge of the frequency and range of situations where he laughed, and an openness to the possibility that he was interested in and capable of connection.

### Nonspeaking autism

In the study reported here, we focused on mothers whose autistic children have limited ability to communicate using speech. It is estimated that about one-third of autistic children and adults are nonspeaking [30]. (In keeping with the preferences of the mothers we interviewed, we refer to their children as “nonspeaking,” but they are also referred to in the literature as “minimally verbal” and “nonverbal” [31]). The target children in our sample represented a heterogeneous group: Some did not use any words; others could say a few words, repeat previously heard utterances, or were learning to use augmentative and alternative communication (AAC) systems. But the focus of the current work was on the mothers’ experience of connection prior to their children having an effective language-based way to express themselves. We recognize the problematic nature of the term “language-based” given debate around what constitutes “language,” “speaking,” and “talking” [32]. We describe the target children as not having access to an effective “language-based way to express themselves” to mean that they did not have a conventional symbolic way to communicate effectively.

We focused on mothers of nonspeaking autistic children for two reasons. First, nonspeaking children cannot easily convey thoughts or beliefs, reminisce, or communicate about the future—common ways that other people connect with each other. We were interested in how these mothers nevertheless come to experience a sense of connection with them. Second, nonspeaking children cannot elaborate on or easily correct interpretations that others make about the meaning of their behavior. It is not easy to know whether a behavior that gets interpreted as a bid for connection, for example, actually was intended as a bid for connection. We were interested in how mothers navigate the uncertainty that arises from making rich, connection-related interpretations of behavior and having few, if any, immediate and reliable ways to know whether those interpretations are correct.

### Guiding questions

Our guiding questions were: How do these mothers experience connection with their nonspeaking autistic children? What child behaviors did they see as contributing to it? What convictions are associated with it?.

## Materials and methods

This interview study was performed and is reported in accordance with the standards for reporting qualitative research (SRQR) checklist [33]. The study was reviewed and approved by the University of California, Santa Cruz Institutional Review Board (Protocol #2387) and the University of Virginia Institutional Review Board for the Social and Behavioral Sciences (Protocol #2342). The participants provided written informed consent prior to their participation and were informed that the interviews would be audio recorded. They were assured via the written consent procedure and verbally at the time of the interview that their participation would be confidential; aliases are used below.

### Researchers

The research team comprised two developmental psychologists (one of whom is a parent of a nonspeaking autistic child who was 7 to 11 years old during the course of this project); one philosopher; and one anthropologist (who served as the interviewer and is the parent of a nonspeaking autistic child who was 13 to 17 years old during the course of this project).

### Participants

The 13 participants were mothers who met two criteria: a) had a nonspeaking autistic child at least 5 years of age, and b) had blogged or publicly spoken about their positive experiences (as well as challenges) parenting that child. We focused on mothers with this profile for two reasons. First, it was important that the target children were old enough that they would be expected to talk. Second, because we were interested in understanding the experiences of mothers who felt connected to their children, it was important that we recruit participants who we knew felt this way. The interviewer initially contacted 10 mothers she knew through their online writing and who met these criteria; they recommended three additional mothers. Although fathers were invited to participate, ultimately it was 13 mothers who committed to the interview process.

Profiles of some of the mothers are provided in a section below. Here, we offer some general demographics. Mothers ranged in age from 35 to 52 years (average: 44 years). Eleven of the 13 mothers were married or living with a partner. At the time of the interview, they were living in the U.S. (11) or Canada (2). All had a college degree; 10 additionally had a graduate degree. Reported household incomes fell into the ranges $20,000–50,000 (2), $50,000–80,000 (2), $80,000–100,000 (2), and over $100,000 (7). The racial/ethnic makeup of the sample was 10 White, 2 Asian, and 1 Hispanic/Latino. One mother disclosed that she was autistic; another disclosed that she was gay.

### Target children

Of the nonspeaking autistic children described by the mothers, 10 were boys (ages 8 to 14 years), and 3 were girls (ages 5 to 9 years). The age of the autism diagnosis ranged from 18 months to 3 years; all children had been diagnosed at least two years prior to their mother’s interview. Many of the children had co-occurring conditions, including ADHD, anxiety, polymicrogyria, seizures, and apraxia. Nine were enrolled in public schools, two in private, and two were home-schooled.

Seven children (5 boys, 2 girls) were only children, three (2 boys, 1 girl) had both older and younger siblings, one boy had an identical twin, one boy had a younger sibling, and one boy had two younger siblings (one of whom was a speaking autistic child).

### Data collection

We used a phenomenological interviewing technique because it is designed to encourage interviewees to share their lived experiences and how they assign meaning to those experiences [34]. We prepared a script of open-ended questions and potential follow-up questions (see Table 1). But, in keeping with the phenomenological approach, the interviewer flexibly followed up on participants’ responses. These follow-up questions allowed the interviewer to build upon, explore, and clarify the categories of meanings offered by participants, mainly by eliciting specific examples. Because we were interested in how the mothers experienced what they considered to be connection, we did not offer a definition of it, nor did we provide examples of it.

**Table 1:** Parent interview guide.

• Tell me a little bit about your child and how you would describe your relationship with him/her.
• Can you give me some examples of ways that your child communicates that might only be understood by you within the context of your relationship? Has your understanding of that changed over time?
• If I were with your child, would I understand his/her attempts to communicate?
• Were there any pivotal, aha moments that changed your understanding of your child and how he/she was communicating?
• How do you characterize your child’s speaking ability?
• What kind of relationship does your child have with the other important people in his/her life? What does their communication look like?
• When people don’t develop a good channel of communication with your child, do you have thoughts about why that is?
• How did getting a diagnosis of autism change your relationship with your child or your perception of your child?
• Did the diagnosis change how you communicated (also behavior) with your child? What did your communication (also behavior) look like before/after the diagnosis?
• If the subject of experts comes up: How did that change/affect your parenting?
• Give an example of something your child notices that you may not.
• Describe a moment or moments when you feel very connected to your child.
• How is your relationship with your autistic child different from your relationship with your other children?
• What advice would you give other parents?

It is important to note that in our recruitment materials and prior to the interview, we made it clear that the focus of the interview would be on what led mothers to feel a sense of connection with their child: “Our research focuses on factors that contribute to a positive relationship between a nonspeaking autistic child and his/her parents. As you know, so much of the research focuses on the stresses involved in parenting a nonspeaking autistic child. Without denying or minimizing the stresses, we’re interested in exploring the positive aspects of these relationships.” Thus, while the mothers were free to raise whatever issues they wished during the interviews, they knew from the outset of our interest in positive aspects of their relationship with their child. Furthermore, as noted, we recruited mothers we had reason to believe (by virtue of topics they had publicly written or spoken about) felt it important to emphasize those positive aspects.

A single, private interview lasting between 45 and 90 minutes was conducted with each participant at a time of their choosing, via Facetime or telephone, and was audio recorded. All interviews were conducted by CS, who disclosed that she was the mother of a nonspeaking autistic child in order to establish rapport with interviewees and to demonstrate her familiarity with some of the issues likely to arise during the conversation. Mothers were compensated $50 for their participation.

### Data analysis

The interviews were transcribed verbatim by a professional transcription service. The accuracy of each transcript was verified by at least two members of the research team, who listened to the recordings while proofing the transcripts; any disagreements were resolved through discussion. Thematic analysis was conducted following the recommendations of Braun and Clarke [35]: The transcripts were read repeatedly by all members of the research team; content relevant to the mothers’ experience of social connection was coded, discussed, and grouped into candidate themes and sub-themes using Dedoose software. Once candidate themes and sub-themes were identified, transcripts were re-read by members of the research team. New themes and sub-themes were added to accommodate previously uncoded data, existing themes and sub-themes were collapsed or split to more accurately capture the meaning intended by interviewees, and the entire process was repeated. Thematic saturation was considered to have occurred when additional readings of the transcripts did not result in changes to the previous round of coding. Final themes and sub-themes were reviewed and refined by VKJ and CS through discussion.

## Sample profiles

Before explaining the results of our thematic analysis, we first provide brief narrative profiles of three of the mothers we interviewed. These profiles serve two purposes. First, they describe the familial worlds that some of our interviewees inhabit, and they highlight the powerful social currents mothers of nonspeaking autistic children can face. This helps to provide context for the themes and sub-themes we describe in the Results section. Second, the profiles illustrate some of the personality and experiential factors that were not the focus of our interviews and so are not reflected in our thematic analysis, but which may have influenced how these mothers came to feel strongly connected to their nonspeaking autistic children.

### Lucy

Lucy’s only child, John, was born three months early and diagnosed with multiple disabilities (he was 12.5 years old at the time of the interview). John was immediately off to a very rough start and was not expected to live. Lucy recounted how a nurse in the NICU where he spent the first seven months of his life told her: “Don’t decorate the nursery just yet.” Although she is not a religious person, the odds against his survival were so great that she describes him as a “miracle.” She and her husband spent those seven months learning how to care for their son, spending long hours at his side in the hospital.

It was during this time that Lucy decided that she could not return to work because the cost of the full-time nursing care that John needed would have exceeded her income. Instead, she focused on becoming the “parent he needed me to be instead of the parent I thought I should be.” At various times, this involved homeschooling John—who started his school life at 2.5 years of age—and managing most of his day-to-day needs and therapies as the primary at-home parent. (Within a few years of John’s birth, her husband went back to school full-time, training for a job in the medical field.) Lucy began writing an online blog about her experience parenting John, allowing her to participate in a public form of discourse to which many mothers had turned. She maintained her blog for nine years (2007–2016).

Lucy had previously studied broadcast journalism, which complemented what she described as a natural ability to pick up on nonverbal cues and body language. In her efforts to become the mother John needed her to be, she describes how she “trained” herself to “try to listen to not just the words on the surface.” At various times in the interview, she describes her many advocacy efforts for her son in the expected realms of school and medicine, but also within her own family, stating:

I had to start fighting every day with people around me, even my own family, about how to talk about my child in a way that was respectful, in a way that was hopeful, in a way that held open the possibilities that we have no idea what is there inside of him.

Asked to describe her son, she said: “He’s smart. He’s sassy. He’s funny.” As we will describe below, she believes her nonspeaking son tells her he loves her 100 different ways every day.

### Diane

Diane and her husband have two children: a nonspeaking autistic son (Juan, 7 years old at the time of the interview) and a non-autistic son (5 years old at the time of the interview). Describing her family, Diane said: “We are a parks and wrestle-at-home family right now,” and noted with delight that one of Juan’s favorite things to do at the park is “to sift wood chips or sand. … and also to move things, so pine needles, and build these giant towers out of these things.” The family had recently moved from the Midwest to the West Coast, leaving behind her husband’s family, as well as the therapies and school supports they had painstakingly set up for Juan. At their new home, they do not have in-person support from either of their families.

Diane is a college-level English instructor. At the time of the interview, she was working as a writer focusing on topics related to autism while also homeschooling Juan and his brother. Diane described her desire to make positive messages about autism more widely available to parents of newly diagnosed children, a topic that she returned to many times during the interview. She recounted how deeply affected she was by what she read shortly after Juan was diagnosed:

The first book I read was *Let Me Hear Your Voice* [by Catherine Maurice], the recovery narrative. … If your point of entry is a recovery narrative, it sets up a false expectation, right?… It just makes you think that if you keep looking hard enough, you'll figure it out. And that's a devastating place to be as a parent … and for your child because nobody can win at that scenario, right?

Like a number of mothers we will quote below, Diane spoke poignantly of how Juan’s diagnosis altered, for a time, how she thought of her son:

When Juan was born, I was just floored by my adoration for this child, right? I mean just all things baby, and like we breastfed him, we co-slept and I just couldn't get enough of him, you know, we went everywhere together.… [O]nce he got his diagnosis, I was very shaken.… [it was like] “Oh I had this kid and then I lost him”.

It took Diane 3–4 years after Juan’s diagnosis before she had what she describes as an epiphany about her own ability to be his “best advocate” by resisting what she was being told about her son by many autism professionals in their life and turning instead to the writings of autistic self-advocates.

### Michelle

Michelle and her husband were relieved when their daughter Rose was diagnosed with autism at two years of age. Following a significant illness, their bilingual child had rapidly lost the ability to speak, and her parents had feared a more catastrophic outcome. Michelle already knew something about autism when her daughter was diagnosed. When Michelle was 14, she had been acquainted with a young autistic boy who had fascinated her, spurring her to read whatever she could on the subject. Of the experience she said:

I really liked to hang out with him, and I always thought there's more than they say in these books.… It's interesting and I think maybe these books got it a little wrong.… I thought that… because I was, I was able to connect with him very well, with this little boy, and I thought maybe it's not as bad as they say. Right?

Michelle reports that she never felt the kind of sadness about Rose’s autism diagnosis that she recognized in the writings of other mothers whose online blogs she was reading. At the time of Rose’s diagnosis, Michelle already had two other children and was expecting her fourth (at the time of the interview, her four children ranged in age from 3 and 13 years old). The experience of parenting her two older children gave Michelle, who was a stay-at-home parent, the framework for understanding how she wanted to parent her autistic daughter. As she explained:

I always [thought] to myself, “Okay,” especially when she was two, I would say, “Would I have done it for my other children, when they were two? Would I have put them through forty hours a week of therapy? Would I be driving them around from place to place when they were two? Or were they home watching *Dora* [*the Explorer*]?” They were not doing all that stuff, right? They were not doing much of anything. So, I think is it really fair for this child who has so many issues already and she has too many appointments and she gets tired easily and you know? … We never thought, oh, it's a race, you know, we have to do it as fast as possible, you know, the windows are closing. We thought, she’s two. Like, you know, if she waits till she's three, it's going to be okay.

Michelle explained that her family is active in the community, the outdoors, and with extended family (who live nearby). But she recognizes that a good balance for Rose (5.5 years old at the time of the interview) means spending a great deal of time in solitary pursuits. Rose occasionally invites Michelle to watch her beloved *Thomas the Tank Engine* videos, invitations that Michelle finds particularly meaningful because she knows how much Rose enjoys watching them on her own. Michelle talked frequently about allowing her daughter the freedom to engage in solitary activities, which (somewhat ironically) have been important to the relationships Rose has developed. For example,

She really seems to enjoy father-in-law a lot. And he's a very quiet man. Like he won't go out of his way to bother her. He will just sit down. And she'll just sit beside him. And they’ll just sit there, and it’s great.

## Results

As noted earlier, we chose not to give the mothers a definition of “connection,” nor did we ask them to define it. But as they were explaining how they experienced connection and as we will see below, the examples the mothers provided focused on the closeness they felt by negotiating understanding of and responding to their child’s feelings and needs, the joy and comfort they felt in being with their child, and their child’s apparent joy and comfort in being with them.

We frame our findings in terms of three themes: 1) a range of child behaviors elicited feelings of connection; 2) mothers (re)framed child behaviors that could undermine connection as caused by factors unrelated to the relationship; and 3) mothers held several convictions that may help create and sustain connection in the face of uncertainty. Note that we will defer detailed discussion of how the themes relate to each other until the Discussion.

### Theme 1: Behavioral categories eliciting feelings of connection

We inductively identified five categories of child behavior—things their children do—that the 13 mothers described as leading them to feel connected to their children. For expository purposes, we describe these five categories separately, but as the examples we provide illustrate, a given situation or moment of connection that the mothers described almost always involved child behaviors from more than one category. Table 2 shows which categories were represented in each mother’s interview.

**Table 2:** Interview themes and sub-themes.

	Theme 1: Behavioral indicators						Theme 2: (Re)framing behavior		Theme 3: Convictions in the face of uncertainty			
Mother (Child; Child’s age)	Speech	Contact/proximity	Responses to being together	Eye contact	Invitations to join activity	Unconventional	Reframe	Provide support	Uncertainty	Personality	Presume competence	Acceptance
Suzanne (Mollie; 7)		✓	✓	✓		✓	✓	✓	✓	✓	✓	✓
Sharon (Patrick; 8)		✓	✓	✓	✓		✓	✓	✓	✓	✓	✓
Sally (Luke; 12)	✓	✓					✓	✓	✓	✓	✓	✓
Ruby (Steve; 13)			✓			✓	✓		✓	✓	✓	✓
Michelle (Rose; 5)		✓	✓	✓	✓	✓			✓	✓	✓	✓
Mary (Peter; 13)		✓	✓	✓			✓	✓	✓	✓	✓	✓
Lucy (John; 12)	✓	✓	✓	✓	✓	✓	✓	✓	✓	✓	✓	✓
Lily (Guy, 14)		✓	✓				✓	✓	✓	✓	✓	✓
Iris (Carlos; 11)	✓	✓	✓	✓		✓	✓	✓	✓	✓	✓	
Diane (Juan, 7)		✓	✓	✓		✓	✓	✓	✓	✓	✓	✓
Cecilia (Paul; 13)		✓	✓	✓			✓	✓	✓	✓	✓	✓
Barbara (Jonas, 11)		✓	✓	✓		✓	✓	✓	✓	✓	✓	✓
Angie (Kate, 9)		✓			✓	✓	✓	✓	✓	✓	✓	✓
Total	3	12	11	9	4	8	12	11	13	13	13	12

*Note*. All names are aliases.

#### The role of (limited) speech

Although the mothers in this sample had children with limited ability to communicate effectively using speech, some children, as noted earlier, could say a few words or used an AAC device. Three mothers explained that they experienced connection on the rare occasions when their children expressed their love through words. For example, when asked to provide a specific recent example of when she felt connected to her child, Iris described how, after a difficult day with her son Carlos, she told him:

You know, I understand you can’t—it’s really hard for you to find the words. But I really need you to come up with one or two words.” And we sat there for, I don’t know, a couple minutes, and he finally came up with some, some random stuff, but it was, it ended with “mom forever”.

Two mothers said they recognized that speech would provide a means of connection if it were consistently available. But not having it available led them to a greater appreciation of other ways to connect, including those described in the sections below. Lucy, the mother profiled earlier who felt her experience in broadcast journalism helped sensitize her to nonverbal communication, said, “I mean sure, I would love nothing more than to hear his own voice say, ‘Mommy, I love you.’ But he tells me ‘Mommy, I love you’ 100 different ways every day.” Similarly, Mary explained that her relationship with her nonspeaking son had taught her that:

words often will take away from being in the moment and being open to that affective, somatic, being in the moment experience with somebody. So, you can really understand a lot of somebody by being in the same room with them.

Indeed, as the next section shows, physical co-presence was a commonly described source of connection for the mothers we interviewed.

#### Connecting through physical contact and proximity

Twelve of the 13 mothers mentioned physical contact—hugs, kisses, cuddles, tickles, roughhousing, and holding hands, for example—as a source of connection. Lily, for example, explained that she experiences connection when her son puts his arm around her shoulders or holds her hand while walking down the street (“It’s less of a security thing than it used to be”).

Six mothers who mentioned physical interaction as a source of connection additionally described experiencing connection in their children’s attempts to be physically close to them, even when the children did not seek out additional interaction. For example, Barbara said:

He’ll like scoot up like right next to you when you’re on the couch, and… It gets so funny. Sometimes when he was younger he would do this. He’d sit like right next to you, his body would be right pressed up against you, and he wouldn’t be looking at you or anything or trying to talk to you or otherwise interact with you. It was just, like, you were physically touching, you know.

Similarly, Lucy explained that her son will:

sort of snuggle really close to me. He won’t snuggle, like hug or lay on me, but he just sits, kind of sits, kind of like if you’re trying to seat two people on a really narrow bench. And your thighs kind of overlap, even though there’s like three feet of sofa on the other side of him.

#### Connecting through child responses to being together

Eleven mothers described experiencing connection when their children behaved in ways to suggest they enjoyed being together (e.g., smiling, gaze, kissing, leaning in)—sometimes during a shared activity, sometimes during parallel activities, and sometimes simply when they were together without engaging in any activity. For example, Mary said:

[When] it’s just him and I, we’re just hanging out or we’re just in his bed at night reading a book or whatever, I think those are just… That, those are the moments. Oh gosh, there’s so many you know, so many, and then he just, he will look me in the eye or lean over and smile and give me a kiss, or just lean into me like that with his mouth, and there’s just so many. If one had to list them… of these connected moments, there’s just too many.

Similarly, when elaborating on the “100 different ways” her son shows his love, Lucy reported that when she is cooking in the kitchen and her son is in the playroom, separated by a gate,

if he wants my attention or I turn to talk to him, he’ll come to the gate and sometimes, he’ll just kind of look at me in a sort of a, mmm, beatific smile. Like ‘I know some kind of great secret,’ and I instantly go, ‘Okay, what’s he up to?’ And he’ll just lean in and kiss me.

Seven mothers in addition to Mary and Lucy spontaneously mentioned a sense of connection when their child looked them in the eye. For example, Diane, the mother profiled earlier who felt “shaken” after her son’s diagnosis, explained that eye contact is one powerful way she experiences connection with him now: He will “peer intently at you, and you know, it can be something as simple as I wanna take a shower or go to the bathroom and that’s kind of the context, but it’s just this look of understanding and connection.” She explained that she and her husband even joked that their son’s eye contact was a “‘look into your soul’ look”.

As noted in the Introduction, eye contact is often interpreted among non-autistic people in Western cultures as a sign of social interest, but autistic people engage in it much less often—indeed, lack of eye contact is among the behaviors that clinicians use when diagnosing autism [6]. Sharon explained that she appreciated when her son made eye contact all the more because it was relatively infrequent: “I recognize that I still have those traditional notions of, you know, how you relate to people, but I do appreciate when he looks me in the eye and he smiles. Those are few but precious moments for me”.

#### Connecting by being invited to join

Four mothers described feeling connected when their children invited them to join in an activity, which is often interpreted in typical development as a bid for joint attention [36]. For example, Lucy explained a moment of connection when her son, whom she describes as “obsessed” with letters, guided her hand to trace letters. Michelle, the profiled mother whose bilingual daughter suddenly lost the ability to speak at two years of age, described the connection she felt when her daughter invited her to watch favorite *Thomas* videos—even though Michelle knows that she enjoys watching them on her own:

She will pull us to watch it with her, and to show us moments that she seems to like, and she will watch our face for a reaction. Then, she’s laughing, like, “Isn’t this the best thing you’ve ever seen?” And she will keep rewinding it.

#### Connecting in unconventional ways

Eight mothers also described unconventional things their children did that led them to feel connected. Barbara explained that when she gets home from work, her son sometimes insists that she make trips to the kitchen to get food for him—a behavior that she interprets as his attempt to affirm their relationship after her absence:

In some weird way, it’s like I’m going to, you know, I’m going to order mom around and I’m going to like request things and make her go to the kitchen and get me stuff. And that’s how I know that she loves me, or I don’t know. That’s how, you know, that’s h-, that’s what we do. And so I think it maybe, you know, kind of, he feels like it’s reinforcing a bond between us, you know, to do that.

Angie explained that after helping her daughter to persevere through a difficult event, her daughter:

will take my hand and put it over her mouth and give me a kiss on my hand. And it’s like it’s a gesture that she very specifically does when she’s like “thank you,”… she doesn’t do it that much in other contexts.

### Theme 2: (Re)framing behaviors that could undermine connection

When someone acts in a way that inconveniences other people and there is no way the person can explain why they are behaving in that way, it would be easy to assume that the behavior reflects disregard for those other people. As Barbara put it, “[I]f somebody doesn’t talk and doesn’t, you know, kind of advocate for themselves, like explain why they’re acting a certain way, then we tend to assume the worst.” For 12 of the 13 mothers in this sample, however, challenging behaviors were seen as occurring because of something external to the relationship—difficulties with self-regulation, communication, or anxiety, for example. (The thirteenth mother did not describe any challenging behaviors during her interview.) That is, whereas the child behaviors described in Theme 1 were interpreted as intentional acts very relevant to—even constitutive of—connection, challenging behaviors were interpreted as outside their child’s control and therefore not relevant to connection.

This framing of challenging behaviors, which could happen either in the moment or in retrospect (hence the title of this section, “(re)framing”), had two effects: 1) It helped to prevent the mothers’ sense of connection from being undermined, and 2) it allowed them to better provide the kind of support their children needed, which they felt strengthened their connection. Table 2 shows which mothers described each of these effects.

#### (Re)framing behaviors so they do not undermine connection

Cecilia explained that her son sometimes does things that could be interpreted as uncaring, but she has come to see these as impulsive rather than intentional behaviors:

[T]here were so many things where it made it really hard to say, “Okay, we’re connected,” because you just flushed down my, you know, wedding anniversary present. It’s hard to go like, “Okay, this kid really is connected to me and cares for me,” when stuff like that happens.… [T]he reality is that impulsive behavior can be read by us as uncaring behavior.

Barbara described a period of time where her son had been “really cranky” and had engaged in “a lot of [challenging] behaviors” that made her family’s life difficult. When she discovered that he had a cracked tooth, she realized that the pain and difficulty letting her know its source (rather than disregard for how his challenging behaviors affected others) had led him to act out: “[E]verything he does, he does for a reason. And that even when you, you know, you think, you’re tempted to think he’s just acting out or … there’s always, you know, always look for the reason behind the behavior.” By emphasizing the need to look for the reason behind a behavior, Barbara is recommending engaging in the kind of rich interpretation described in the Introduction—that is, interpretation of a behavior that goes beyond that particular behavior itself, and is informed by the mother’s perspective (see Theme 3 below), history with the child, and understanding of the context.

#### Providing support can elicit feelings of connection

Eleven mothers explained that their dogged pursuit of the meaning behind their children’s challenging behaviors allowed them to provide the kind of support their children needed. For example, Sally’s son sometimes refused to attend his sister’s gymnastics competitions, which had initially seemed “selfish” to her. Before understanding that the venue where those meets were held, the noise level, and the number of people around were overwhelming for him, Sally would have insisted that he attend, which would presumably have led to more protest from her son and more frustration for Sally.

[B]efore I wouldn’t know, “Oh why don’t you want to go and watch your sister’s meet?” But now if I see him hesitate, like his body will just kind of get stiff, I know it’s like too [much] sensory overload or it’s too overwhelming whereas before I would have been like “Come on, just go, stop giving me a hard time.” But now it’s just like “Oh, you know, I understand.… I can understand. Why don’t we, why don’t we go watch from the TV instead? Or why don’t we just hang out here in the lobby until you feel a little more prepared?… Or let’s go take a walk and see if we can come back and try again”.

Mothers believed that providing appropriate support in the face of a challenging behavior had both tangible and intangible benefits: It decreased the challenging behavior, and it strengthened connection by signaling to their children understanding and respect. For example, Iris described a bath time episode where her son was extremely upset, and she could not figure out how to console him. He repeated a line from a song in a Muppet movie (“How could something so right be so wrong?”) until her “sleuthing,” as she called it, led her to realize that he was using that line as a means of indicating that he was upset about her impending divorce. Iris then used it as an opportunity to talk with him about the divorce, which he seemed to appreciate:

And I said, “Carlos, are you thinking about Mommy and Daddy’s divorce?” And he sat straight up and he said, “Yes.” And he made eye contact, and I said, “Do you need me to talk to you a little bit about the divorce?” And he said, “Yes.” And he was completely engaged.

### Theme 3: Convictions that may support connection in the face of uncertainty

Because their children cannot easily confirm interpretations of their behavior, all 13 mothers recognized that the interpretations they made could be wrong. For example, Michelle said of her daughter:

[S]he’ll come and kind of rub our hands or rub our arms because she likes to rub our arm a little bit. And you know, is it for her or is it for us? We don’t really know, but we take it as, you know, connection, right? Something like, she’s saying, “Hey, I know you’re here,” right?

Mothers noted their uncertainty in a variety of ways. For example, when Barbara interpreted her son’s ordering her to fetch him food as connection-driven, she qualified it with “I don’t know” and “maybe.” Similarly, Mary explained that she generally felt the interpretations she made of her son’s behavior were accurate, “[b]ut to say that absolutely? I can never know, right? I can’t.” Sharon explained that after doing something funny, her son sometimes did not show the expected emotion on his face, which she speculated meant he had moved on to something else, “but I have no idea, you know?” Michelle noted something similar, explaining that interpretations of her daughter’s emotional displays were not straight-forward: When she looks content, is she “happy because she went somewhere with us, you know? Or is she happy that she’s going home and it’s over? You know, like we don’t know.”

The uncertainty about whether their interpretations were correct sometimes led mothers to wonder if they were as connected with their children as they thought they were. As Cecilia put it, “[Y]ou can get, like, have those weak moments where you think, ‘Am I kidding myself? Are we really connected?’” And yet despite moments of doubt, the mothers in this sample expressed confidence that they *were* connected and that their children *were* interested in connecting with them. Sharon and Suzanne independently described this belief as “a leap of faith.” As Sharon put it, “[I]f you don’t have a reliable way to know whether or not your child understands you, or whether they’re taking it in… It’s not easy.”

We identified three convictions that, we speculate, may have helped mothers make and maintain this “leap of faith:” The mothers explained that their children 1) have personalities, 2) are more competent than they appear to others, and 3) should be accepted for who they are. Mothers did not always articulate these convictions in the context of connection, but they are relevant to connection because they represent the mothers’ general approach to understanding and interacting with their children. Table 2 shows which convictions were represented in each interview.

#### Attributing personality

All 13 mothers in this sample described their children as full of personality—preferences, attitudes, character traits, and perspectives that persist across space and time. This was evident in the trait adjectives they used when talking about their children, including “smart,” “thoughtful,” “sensitive,” “kind,” “determined,” “laid back,” “sunny,” “sweet,” “friendly,” “joyful,” “charming,” “sassy,” “funny,” “easygoing,” “intelligent,” and “affectionate.”

This is noteworthy because, as three mothers explained, autistic people are not always seen as having personalities. For example, Lily said, “I think that’s something that people just really don’t consider, that autistic people have personalities you know. They’re not just autistic, they’re people! Personalities!” Sharon explained that her son was “like, you know, any child who has emotions and feelings and desires and I don’t know—I think that gets lost, or you know [people] are traditionally trained and see kids with autism not really [having those things].” One reason the mothers in our sample were confident in attributing personality characteristics to their children is because they had seen their children over time and in multiple contexts. As Mary explained about her daughter, “we can know things about her that other people may not know.”

A consequence of attributing a personality to someone is that it provides a lens through which to interpret their behavior. For example, if one believes that a desire for social connection is part of their child’s personality, then behaviors that are less conventional examples of that desire may be interpreted in that way, which will reinforce the belief that a desire for social connection is part of their child’s personality. For example, Sharon, who described her son as “social” and “affectionate,” explained:

So I had to, kind of, basically think of the way that typical people interact, or connect, is not the way that my son does, and I had to really reframe or re-calibrate the way I see that, you know, how people connect. So that I would understand that he was, when he sits next to me he wants to be near me, or when I want to give him a kiss, he doesn’t really kiss me back, but he leans in. He’ll lean in and give me his forehead and then I’ll kiss him.

#### Presuming competence

All 13 mothers noted that it is easy to underestimate a nonspeaking autistic person. As Sharon said, “When you see a child who can’t talk and who’s flapping their hands and jumping up and down? You think this person may not have the same intentions or thoughts or reflections as a typical person.” To counteract this assumption (which they considered inaccurate), the mothers described the importance of “presuming competence” in their children. In brief, “presuming competence” reflects a belief that it is less dangerous to presume that someone has a competence (or the potential to develop that competence given the right kind of support) than to presume the opposite [37]. Lucy, the profiled mother whose son was in the NICU for seven months, articulated why she insists that others (teachers, therapists, members of her own family) presume competence in him, stating that “we have no idea what is there inside of him.”

The mothers mentioned presuming competence most often in the context of presuming that their children understood what they heard. Ruby said: “[I]t’s hard to remember sometimes when you’re in a room with somebody who’s not talking back that they probably understand everything that you say.” For Lily, presuming her son understands what he hears helps her appreciate his empathetic nature and caring disposition:

[H]e understands everything everybody says and it’s really very clear by the way he reacts to everything. And so I think that part of his, his empathetic nature is that he really is watching and listening and observing the whole time because he’s, you know, he’s a very caring individual.

Presuming that someone understands what you say is a prerequisite to perceiving their subsequent behavior as a meaningful response to what you have said, which is part of how rapport is built between people [38]. Suzanne noted how positively her daughter responded to being talked to in an age-appropriate way:

[W]e would, you know, say things that, that are appropriate to say to 5- and 6-year-old girls. And we would just see her whole, her whole body would just change. She would stand up straighter and you know, immediately react to what we had said in a way that there’s no way that could have happened spontaneously, you know. I mean it’s just clearly reacting to what we had just said.

#### Acceptance

Twelve of the 13 mothers explained that accepting their children’s autism ensured that they focused on forging a close relationship rather than on trying to change them. (The thirteenth mother did not discuss her own acceptance of her son’s autism, though she did discuss the importance of her son accepting himself.) They felt that this signaled to their children that their love was not conditional on being or appearing non-autistic. Angie drew parallels between her own experiences with being accepted as a gay person and trying to create conditions where her daughter would be accepted:

[Parents of gay children say] “We’re upset that you’re gay because of the way of the world is going to treat you,” [which] is actually something that repeats itself in being an autism parent. Like “We accept you in the home but there’s this big bad world that doesn’t accept you”… I mean that was a really unconvincing argument to me when I was a, you know, young [gay] person… [I]t’s not that I’m like in a fantasy world about how great the world is about disability. Nonetheless, that’s a really unconvincing—it doesn’t, that doesn’t feel like love to the child, I don’t think.

Sharon described being profoundly influenced in her own process of accepting her son’s autism by an autistic blogger, who wrote about how traditional messages about autistic people suggest they are “impaired” and “broken,” and much of the discourse from scientists and professionals is about how to “fix” them. The blogger explained that autistic people themselves are listening to these “hurtful” messages (see previous section about presuming competence), which can lead to low self-esteem and depression (see also [29]). Sharon (and other mothers) emphasized that accepting her son’s autism did not mean she stopped trying to find ways to support him: “I think parents need to learn to accept their child and still, you know, of course find ways to support and provide the necessary resources for them.”

## Discussion

Mothers of nonspeaking autistic children could face a number of challenges connecting with their children. First, autistic children do not consistently behave in conventional socially oriented ways [1–3,6], and so they might not appear to want to connect. Second, someone who does not have an effective language-based way to express themselves cannot easily share their thoughts, beliefs, or feelings—a common way most other people connect. Finally, mothers cannot readily confirm that the interpretations they make of their children’s behavior—including interpretations about behavior they see as relevant to connection—are correct.

Despite these potential obstacles, the mothers in this sample had each participated in public discourse about the strong sense of connection they felt with their nonspeaking autistic child. The goal of this research was to try to understand how they experienced connection. We found that the mothers interpreted a range of child behaviors as relevant to connection, (re)framed behaviors that could undermine it, and shared a number of convictions that may promote and sustain feelings of connection in the face of uncertainty. It is striking that we identified common sources of connection from these 13 very different mothers reporting on 13 very different children. At the same time, an important contribution of this study is highlighting that a range of behaviors can lead to a sense of connection. In what follows, we discuss and expand on each of these findings.

### Connection-relevant and connection-irrelevant behaviors

We embarked on this project expecting that the mothers might focus on unconventional ways their children showed a desire to connect—inexplicable laughter, for example, as Senator [11] recounted in her memoir. But many of the child behaviors the mothers described were exemplars of more conventional and well-studied categories of social behavior. For example, psychologists have long known that physical contact [39] and close proximity [40] can elicit feelings of connection, as was evident in Lucy’s description of her son’s attempts to snuggle close to her. Contingent behaviors that make it seem like someone enjoys being with you—when someone gazes and smiles at you, for example—have been linked to feelings of closeness [41,42], as when Diane explained her son’s eye contact as a “look of understanding and connection.” And nonverbal invitations to join in an activity, as when Michelle’s daughter invited her to watch *Thomas* videos, are a prototypical way that joint attention is established [36]. While joint attention is often studied and discussed as a tool for learning [43], it is also a means of connection. Sharing an experience with someone can evoke positive emotions that affect how you interact with them, which then affects how they interact with you, and so on [38].

The mothers did provide some examples of child behaviors they interpreted as related to connection that an unfamiliar observer might not interpret in that way. However, as with Senator’s [11] example involving her son’s inexplicable laughter, once they explained the behavior’s history and context (i.e., explained their rich interpretation) it was not difficult to understand why they saw them as related to connection. For example, recall that Barbara’s son sometimes engaged her in a particular activity when she came home from work, ordering her to fetch things for him from the kitchen. She interpreted his behavior as a bid for connection because, as she put it, “I think… he feels like it’s reinforcing a bond between us.” The activity has become a ritual for Barbara and her son—a patterned activity they enjoy doing together. Rituals are a common means by which two or more people (e.g., parents and children, spouses, colleagues) develop and sustain relationships, with each person relying on the other to carry out a particular role for the ritual to be successful [44].

At the same time that they interpreted a range of child behaviors as relevant to connection, the mothers chose to interpret some child behaviors as *not* relevant to connection. For example, Cecilia pointed out that impulsive behavior, common among autistic children, “can be read by us as uncaring behavior,” which can undermine feelings of connection. But the mothers in our sample did not read challenging behaviors as uncaring. Instead, they came to see them as caused by factors like pain, anxiety, difficulties with self-regulation, or sensory over-stimulation. This understanding allowed mothers to provide the kinds of practical and emotional support their children needed. When their children responded positively to this support (e.g., ceasing the challenging behavior, sitting up straighter, saying “thanks”), it actually strengthened the connection the mothers felt because they took that as a sign that their caregiving acts had been successful [45].

### How a diagnosis can undermine convictions supporting connection

As we noted above, the mothers explained that their children had personalities, were more competent than they appeared, and should be accepted for who they are. We suggested that these convictions may have helped the mothers build and sustain feelings of connection with their children even in the face of uncertainty. Ideally, all caregivers would hold these convictions about their children. But the mothers in our sample explained that these convictions are easily undermined by the dominant popular and scientific narrative about autism, which as Gernsbacher [46] pointed out, otherizes autistic people by focusing on how they lack abilities and motivations thought to be central to being human—for example, asserting that they have “deficits in social-emotional reciprocity” [6]. Indeed, 10 of the 13 mothers said that receiving the autism diagnosis negatively affected their ability to feel connected to their child.

For example, as we noted in her profile above, after her son’s diagnosis, Diane doubted the sociable personality she had attributed to him and the feeling of connection she had established with him pre-diagnosis:

I felt like everything I had known about him I didn’t know anymore, and everything that we’d had… like, “Oh I had this kid and then I lost him.” If you’re listening to that [narrative about autism] then… then it’s, it’s very easy to feel like you’ve lost that relationship, right? Or you’ve even lost that child.

Sally shared that when her son was diagnosed with autism, she assumed that he would not be able to connect with her until he was no longer autistic. Rather than presuming that he could connect, she put her efforts into trying to change him (an approach that Lily referred to as “child as project”):

I feel like I shouldn’t have got that diagnosis because I felt that the reason why he couldn’t connect was the autism. And so, the autism had to be eradicated so that he could be, so he could connect. So I [did not] feel like the solution was trying to connect with him. I thought the solution was trying to cure the autism if that makes sense.

Diane noted that an additional consequence of her son’s diagnosis was that she stopped engaging in rich interpretation of his behaviors: Instead of seeing his jumping and spinning as indicative of a “wild mischievous boy who wanted to have fun,” she saw his unusual behaviors as “aberrant,” as something to be eliminated.

For the 10 mothers who felt their sense of connection was negatively affected by their child’s diagnosis, there was no single event that led to the strong feelings of connection they described at the time of the interview. Rather, it was a gradual process that involved getting to know their children and coming to perceive their social behaviors as on a continuum—from conventional (but perhaps infrequent) behaviors that required little in the way of rich interpretation (e.g., eye contact) to less conventional ones that were recognized only in the light of the context and knowledge of their children’s personality and history. In addition, 10 mothers mentioned the profound impact that reading autistic authors and getting to know autistic people had on counteracting stereotypes about autism to which they had been exposed, including the stereotype of autistic aloneness.

### Perceiving social interest in autistic children

Our findings could be considered, at once, unremarkable and remarkable. They are unremarkable in that they show that the mothers’ sense of connection with their nonspeaking autistic children was based, in large part, on the same categories of child behavior that have long been thought to signal social interest. At the same time, the findings are remarkable because one of the criteria for autism is “persistent deficits in social communication and social interaction” [6], and a great deal of research has shown that autistic children do not consistently act in conventional socially interested ways [1–3]. In other words, the mothers described experiencing connection when their children acted in ways that autistic children have not traditionally been thought to act. How should we make sense of this?

One possibility is that the children that the mothers in this study felt connected to were outliers—children who, despite their autism diagnosis, frequently act in conventional socially interested ways. Perhaps they represent a subcategory of autism similar to one identified by Wing and Gould [47], who described a very small group of autistic children that observers indicated “used eye contact, facial expressions, and gesture to indicate interest and to try to join in conversation as best they could” and who “enjoyed social contact for its own sake with adults and with other children” (p. 15). Ours was an interview rather than observational study, so we cannot rule out this possibility.

Another possibility is that some mothers relied more on the fact that there were conventional social behaviors in their children’s repertoire rather than on how often they occurred. Recall, for example, how Sharon explained that her son rarely made eye contact, but that made the occasions when he did make eye contact especially powerful demonstrations of his desire to connect with her. Sharon’s interpretation is interesting because it contrasts with what seems to be an assumption among autism scientists and practitioners that the more someone engages in conventional socially oriented behaviors, the more socially interested they are. (This assumption seems consistent with a more general belief that if a little of something is good for you, a lot of it is better; see, for example, [48].) At the same time, some mothers did feel like their children frequently engaged in connection-oriented behaviors. Recall that Lucy, for example, explained that her son shows her he loves her “100 different ways every day,” and Mary explained there were “just too many” moments of connection to be able to list them all.

Another possible reason the mothers in this study experienced a strong sense of connection is that they expanded the categories of socially oriented behavior beyond the typical, conventional exemplars (see also [41]). For example, we noted above that just four mothers described experiencing a sense of connection when their child invited them to join an activity they could do together—a prototypical way that joint attention is described and studied [36]. But some mothers described experiencing connection when they shared everyday experiences with their child, even if it did not involve overt invitations to join an activity or regular monitoring of each other’s attention. As Lily explained:

We do a lot of hiking together, we do a lot of just hanging out and watching TV together, you know we do cooking, we do chores, those kinds of things, swimming, that kind of stuff. You know we run errands—he’s really, he’s a better sport about it than his sisters are, you know, about just the things that need to be done. And you know we read a lot of books together.

Finally, the mothers in this study recognized that their children’s responses to the mothers’ own bids for connection could be atypical but still indicate their child was reciprocating. For Sharon, for example, this involved adjusting her expectations about ways her son may reciprocate:

So I had to, kind of, basically think of the way that typical people interact, or connect, is not the way that my son does… when I want to give him a kiss, he doesn’t really kiss me back, but he leans in. He’ll lean in and give me his forehead and then I’ll kiss him.

### Limitations and future directions

As with any study, ours has a number of limitations. First, our sample was, by design, unique. Our goal was to understand factors contributing to the positive sense of connection some mothers feel with their nonspeaking autistic children. Because the mothers we interviewed had all publicly written or spoken about their experiences, some of the mothers knew each other and had been influenced by each other’s writings and perspectives. It is not possible to generalize from their experiences to other mothers of nonspeaking or speaking children, to mothers who do not feel connected, or to fathers or other caregivers. Indeed, had we interviewed this same sample at a different point in time, our findings would have been different: As noted, most of the mothers said that their sense of connection had fluctuated over time. Important directions for future work will be to focus on the experiences of other caregivers and to focus more explicitly than we were able to do here on the process by which parents’ sense of connection with their autistic children changes over time (see, for example, [41]). The example profiles we provided earlier offer some hints about the range of personality and experiential factors that may play a role (e.g., dissatisfaction with standard accounts of autism in the case of Michelle, frustration with the recovery narrative for Diane, a sensitivity to nonverbal signals in Lucy, and exposure to and participation in public discourse about autism that was strength- rather than deficit-focused for all of the mothers).

Second, our ability as non-autistic researchers to recognize unconventional categories of social behavior is constrained by our familiarity with the conventional categories. Autistic children may have and develop altogether different ways of connecting with other people (for research on this topic with autistic adults, see [49]). Importantly, however, our data comprised testimony from mothers about the child behaviors they felt were relevant to connection, and our analysis of these behaviors relied on the interpretations the mothers made. This is because our interest was in understanding what their children did that made them feel a sense of connection, not in what the children intended or in what a third-party observer might infer. Our sample had only one autistic mother, and we did not identify any obvious differences between the kinds of social behaviors she described and the kinds described by the non-autistic mothers. A fascinating question for future research would be to ask autistic mothers to reflect on their experiences of connection with their autistic children; additional categories of behaviors and beliefs that can yield a sense of connection may emerge.

Third, we focused on the feelings of connection that the mothers reported with their nonspeaking child. We did not address how that relationship affected (or was affected by) their relationships with other members of their family or others. This would also be a fruitful area of research. Additionally, we obtained only a one-sided understanding of mother-child relationships. Ideally, we would also investigate the children’s sense of connection with their mothers. Studying this question in nonspeaking autistic children presents obvious challenges. However, using observational methods and analytical tools designed to study close relationships in social psychology [50] could allow us to learn about the reciprocal nature of the relationship—how a mother’s interpretation of a child’s behavior influences the mother’s behavior, and how the mother’s behavior then influences the child’s behavior, and so on.

## Conclusions

It is unlikely that you can feel connected to someone if you believe that they are indifferent to you. Scientific and popular narratives often portray autistic children as socially indifferent. But the mothers in this study believed that their nonspeaking autistic children *were* motivated to connect with them. The mothers looked for and found evidence of this motivation in a variety of their children’s behaviors. Of course, looking for and finding evidence of something is a classic example of the confirmation bias—seeing what one wants to see—which is typically considered to be an undesirable approach to gathering objective data. Ignoring evidence that could disconfirm a belief is also a feature of the confirmation bias; when mothers (re)framed behaviors that could undermine connection as caused by factors unrelated to the relationship, this could also be considered an example of the confirmation bias.

But social behavior is behavior that is *perceived* to be social; what counts as social behavior is, by its nature, subjective (i.e., dependent on individual subjects and their points of view). For example, a mother may perceive her nonspeaking autistic child’s fleeting glance toward her as an indication of their social interest, and you may not. Without a way to confirm with the child, there is no way to know whose interpretation is correct. The mothers in our sample recognized (and occasionally felt) this uncertainty. But they regarded their children as interested in connecting with them, and they perceived and experienced their children’s behaviors as connection-driven.
